# Screening and brief intervention for low risk drug use in primary care: a pilot randomized trial

**DOI:** 10.1186/1940-0640-10-S2-O45

**Published:** 2015-09-24

**Authors:** Richard Saitz, Seville M Meli, Tibor P Palfai, Debbie Cheng, Daniel P Alford, Judith A Bernstein, Jeffrey H Samet, Christine A Lloyd-Travaglini, Christine E Chaisson

**Affiliations:** 1 Community Health Sciences and General Internal Medicine, Boston University Schools of Public Health & Medicine/Boston Medical Center, Boston, USA; 2 Community Health Sciences, Boston University Schools of Public Health & Medicine/Boston Medical Center, Boston, USA; 3 Psychology and Brain Sciences, Boston University College of Arts and Sciences, Boston, USA; 4 Biostatistics and General Internal Medicine, Boston University Schools of Public Health & Medicine/Boston Medical Center, Boston, USA; 5 General Internal Medicine, Boston University School of Medicine/Boston Medical Center, Boston, USA; 6 General Internal Medicine and Community Health Sciences, Boston University Schools of Public Health & Medicine/Boston Medical Center, Boston, USA; 7 Data Coordinating Center, Boston University School of Public Health, Boston, USA

## Background

Universal screening and brief intervention (SBI) for drug use among primary care (PC) patients lacks efficacy but the efficacy of SBI for low risk drug use is unknown. This 3-arm pilot study tested the efficacy of two brief interventions (BIs) for drug use compared to no BI in PC patients with low risk drug use identified by screening.

## Material and methods

We randomly assigned participants identified by screening with Alcohol Smoking and Substance Involvement Screening Test (ASSIST) drug specific scores of 2 or 3 (consistent with low risk drug use) to: no BI, a brief negotiated interview (BNI), or an adaptation of motivational interviewing (MOTIV). BNI was a 10-15 minute structured interview conducted by health educators. MOTIV was ¿45 minutes with an optional booster conducted by trained master's-level counselors. Primary outcome was number of days use of self-identified main drug in the past 30 as determined by validated calendar method at 6 months. Analyses were performed using negative binomial regression adjusted for baseline use and main drug.

## Results

Of 142 eligible adults, 61(43%) consented and were randomized. Participant characteristics were: mean age 41; 54% male; 77% black. Main drug was marijuana 70%, prescription opioid 10%, cocaine 15%; 7% reported injection drug use and mean days use of main drug (of 30) was 3.4. At 6 months, 93% completed follow-up and adjusted mean days use of main drug were 6.4 (no BI) vs 2.1 (BNI) (incidence rate ratio (IRR 0.33, 95% CI 0.15-0.74) and 2.3 (MOTIV) (IRR 0.36, 95% CI 0.15-0.85).

## Conclusions

BI (both BNI and MOTIV) appears to have efficacy for preventing an increase in drug use in primary care patients with low risk use identified by screening. These findings raise the potential that less severe patterns of drug use in PC may be uniquely amenable to brief intervention and warrant replication in a larger trial.

